# Positive Effects of Specific Exercise and Novel Turning-based Treadmill Training on Turning Performance in Individuals with Parkinson’s disease: A Randomized Controlled Trial

**DOI:** 10.1038/srep33242

**Published:** 2016-09-13

**Authors:** Fang-Yu Cheng, Yea-Ru Yang, Li-Mei Chen, Yih-Ru Wu, Shih-Jung Cheng, Ray-Yau Wang

**Affiliations:** 1 Department of Physical Therapy and Assistive Technology, National Yang-Ming University, Taipei, 112, Taiwan; 2 Department of Neurology, Cheng Hsin General Hospital, Taipei, 112, Taiwan; 3 Department of Neuromuscular Disorder, Chang Gung Memorial Hospital, Linkou, 333, Taiwan; 4 Department of Neurology, Mackay Memorial Hospital, Taipei, 104, Taiwan

## Abstract

Two different training strategies to improve turning performance in individuals with Parkinson’s disease (PD) were designed and investigated in this study. Subjects were randomly assigned to a specific exercise group, turning-based training group, or control group to receive training that emphasized balance and strengthening, turning-based treadmill training, and general exercise training, respectively. A total of 12 30-min training sessions followed by 10 min of turning training on a level surface were administered over 4 to 6 weeks. The results (n = 12 for each group) showed that both the specific exercise and turning-based training group experienced improved turning performance, the primary outcome, compared with the control group (specific exercise, 33% change, p = 0.016; turning-based training, 35% change, p = 0.021). For the secondary outcomes, the specific exercise group performed better than the control group on the Tinetti balance scale, limit of stability test and lower extremity extensor and abductor strength. The turning-based training groups performed better than the control group in sensory organization and ankle plantar flexor strength. In summary, specific exercise training and turning-based treadmill training were both effective in improving turning performance in participants with PD. However, the improvements in turning performance of these two groups resulted from improving different aspects of impairment in individuals with PD.

Turning is an advanced walking task that is an essential component of goal-directed locomotion in daily life. Falls while turning are eight times more likely to result in hip fractures compared with regular walking^1^. Parkinson’s disease (PD) is a progressive neurodegenerative disease that can result in difficulties in postural control, gait, and advanced walking tasks. Previous researchers have indicated that more than 50% of people with PD have difficulty in turning that can lead to falls^2^. Individuals with PD turn more slowly, have a narrower base of support, and exhibit increased freezing of gait compared with regular walking. These disturbances not only affect the quality of their turning performance but can also cause falls. A previous study found that exercise training improved mobility and also led to documented neurochemical and neuroplastic changes that occurred after the exercise intervention^3^. However, effective exercise training protocols to improve turning performance have not yet been established.

To our knowledge, only two studies have reported on the effects of exercise training on turning performance in patients with PD. Kurtais *et al*. noted an improved turn time on the U-turn task after 6 weeks of treadmill training, but this improvement was not superior to that of home-mobility exercise^4^. McNeely and Earhart found that a rotating treadmill they designed did not exert beneficial effects on turning performance^5^. Previously, we found that balance ability and lower extremity muscle strength, especially of the extensors and abductors, influenced turning performance in individuals with PD^6^. A review study indicated that progressive resistance training can improve functional mobility in PD patients^7^. It has also been reported that balance and lower extremity strengthening exercises can result in positive effects on gait performance^8^. It is not known whether training strategies that emphasize balance control and lower extremity muscle strength improve turning performance.

However, motor learning involves the repetition of desired movements and training specificity, and “specific and repetitive” training protocols should be encouraged to obtain the most desirable therapeutic effects^9^. For instance, treadmill training is a repeatable and specific training program for gait performance. The conclusion of a Cochrane review identified beneficial effects of treadmill training on gait parameters, including gait velocity, stride, and step length, in patients with PD^10^. Based on this task-specific concept, we designed a rotational treadmill and demonstrated that turning-based treadmill training could improve turning performance in individuals with chronic stroke^11^. Analogous to the specific exercise training mentioned above, a novel turning-based treadmill training designed to improve turning performance was also investigated in this study. The purpose of the present study was to investigate the effects of different training strategies on turning performance in individuals with PD. Because balance and lower extremity resistance training may optimize functional gait performance^8^, and because a task-specific turning training protocol resulted in positive effects on turning performance^11^, we hypothesized that a balance and resistance training regimen and a turning-based treadmill training program could improve turning performance.

## Results

**Figure 1:**
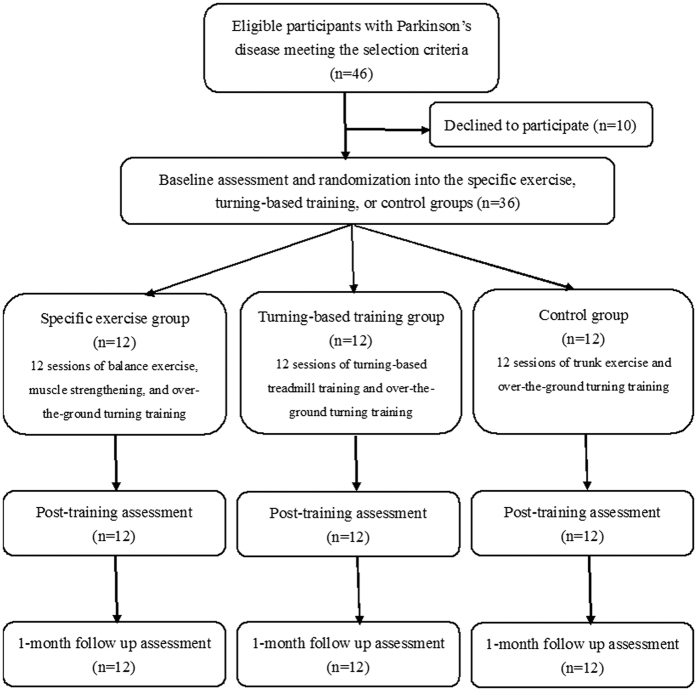
Study flow chart.

Thirty-six participants were randomly assigned to the specific exercise, turning-based training, or control group (n = 12 for each group, Fig. 1). No significant group differences in baseline demographic characteristics or clinical features were found (Table 1). Additionally, no significant group differences were noted in any outcome measures at the pre-intervention assessment (Tables 2, 3, 4, 5). Three subjects in the specific exercise group and two subjects in the control group reported musculoskeletal soreness after the first intervention session. However, the soreness did not persist for more than 48 hours. Three subjects in the turning-based training group reported dizziness during the first session of treatment; however, it subsided within 5 minutes and did not affect their willingness to participate. No other adverse effects were reported during the training periods.

### Turning performance

The turning performance results are shown in Table 2. In the step/quick turn (SQT; Balance Master System, NeuroCom International, Inc., Clackamas, OR, USA), the specific exercise group and turning-based training group showed greater improvements in turn time than the control group after training (specific exercise, p = 0.016; turning-based training, p = 0.021). However, there were no significant differences between the specific exercise and the turning-based training groups. Regarding the performance on the sudden 180° turn during the walking test, no significant group differences were indicated.

### Sensory integration ability

The results of the sensory organization (SOT) test from Balance Master System are shown in Table 3. The turning-based training group showed greater improvements in total score than the control group after training (p = 0.007). Moreover, the turning-based training group also showed greater improvements than the other two groups in vestibular ratio after training (p = 0.019 vs. control group; p = 0.008 vs. specific exercise group), which extended into the follow-up period (p = 0.013 vs. specific exercise group).

### Dynamic balance ability

The results of the Tinetti assessment and limit of stability (LOS) test from Balance Master System are shown in Table 4. Compared with the control group, only the specific exercise group showed improvements in the Tinetti balance score and maximal excursion of the LOS test in the anterior direction after training (Tinetti balance score, p = 0.007 at post-training; maximal excursion, p = 0.023 at follow-up). However, the 360° turn time on the Tinetti scale was decreased to a significantly greater extent in the specific exercise group than in the control group (p = 0.01 at post-training, p = 0.022 at follow-up). These significant decreases in 360° turn time were also observed in the turning-based training group compared with the control group (p = 0.003 at post-training, p = 0.05 at follow-up).

### Muscle strength

The specific exercise group showed greater increases in lower extremity extensor and abductor strength than the other two groups (extensors, p < 0.001 at post-training and follow-up vs. control group, p = 0.013 at post-training vs. turning-based training group; abductors, p < 0.001 at post-training and follow-up vs. control group, p = 0.011 at post-training vs. turning-based training group). The ankle plantar flexor strength of the turning-based training group improved more than in the control group both after training (p = 0.019) and at 1-month follow-up (p = 0.002) (Table 5).

## Discussion

The main finding of this study was that 12 sessions of specific exercise training focusing on dynamic balance and lower extremity muscle strength and turning-based training using a rotational treadmill were both effective in improving turning performance in participants with PD. However, the improvements in these two groups resulted from improvements in different aspects of impairment.

Positive effects of exercise on improving motor control and functional mobility have been documented in patients with PD^12^,^13^,^14^. For example, intensive and challenging task-specific and functional mobility training may increase neuroplasticity in the basal ganglia system, which is responsible for active motor control in PD patients^15^,^16^. Neuroprotective and neurorestorative capacities after intensive exercise have also been reported by animal studies^17^,^18^,^19^. Furthermore, the exercise-induced benefits on overall brain health, including increased blood flow and trophic factors and a stronger immune system, may help address the environmental need for neuroplasticity in the damaged brain^20^,^21^,^22^. Therefore, the improvements in turning performance after training in our study may also be attributed to the mechanisms mentioned above.

Based on the theory of physical therapy, two training strategies are usually used and have been proven to be effective in neural rehabilitation: combined impairment-level and functional activity training and task-specific training. A high correlation between impairment-level training and functional activity has been found^23^. In addition to impairment-level training, functional movement must be included in an intervention program that maximizes training effects. Teixeira-Salmela *et al*. demonstrated that both impairment level (i.e., muscle strength) and deficits in functional mobility (i.e., balance ability) influence gait performance. Accordingly, physical therapists should combine impairment-level and functional mobility training to improve gait performance^24^. Furthermore, our previous study found that balance control ability and lower extremity muscle strength (extensors and abductors) influenced turning performance in individuals with PD^6^.

Based on these findings, one of the treatment strategies in this study was a specific exercise program designed to emphasize balance and muscle strengthening for turning training. The results showed that the turn times on the SQT decreased after this specific training program. Regarding task-specific training, its strategy is to practice functional activities directly to obtain maximal improvement. Most of the current training programs in physical therapy for PD patients focus on this concept to improve functional activities such as transferring, walking, going up/down stairs, and grasping^25^. We thus extended this strategy when designing our program for improving turning activity. Compared with general exercise, the turning-based treadmill training was more effective in improving turning performance, as evidenced by the shorter turn times on the SQT.

In our previous study, we found that patients with PD had a greater fall risk if their SQT turn time was greater than 2.29 sec^6^. Our participants averaged a turn time of more than 2.29 sec at the pre-treatment evaluation. After receiving specific exercise or turning-based treadmill training, the participants’ average turn time decreased significantly (from 2.7 to 1.8 sec and 2.6 to 1.7 sec for specific exercise and turning-based treadmill training, respectively), indicating that their risk of fall may be decreased. Furthermore, the participants’ performance on the 360° turn on the Tinetti assessment scale was also improved, similar to the improvement on the SQT. Although the 360° turn is not a common activity in daily life, the clinical significance of this task cannot be underestimated. According to a previous study, the 360° turn test has good sensitivity for identifying multiple falls in the elderly population^26^. Therefore, the different training strategies proposed by this study appeared to be effective in improving turning performance and may decrease the risk of falls.

Nevertheless, performance on the sudden 180° turn task did not improve significantly. According to the center-surround hypothesis, the excessive inhibition of desired and undesired movements in PD patients, probably because of basal ganglia dysfunction^27^, results in difficulty selecting and executing desired movements. This difficulty has been considered an underlying mechanism for problems in changing motor programs^28^,^29^. Sudden directional change during walking is a complex task that includes receiving an instruction, rapidly modifying the motor plan, and executing the modified program while simultaneously maintaining whole-body balance^30^. Because of their difficulties in unexpectedly changing motor programs, PD patients cannot perform sudden turns easily^31^.

The SQT and 360° turn tasks are relatively easy because the subjects can prepare for the turning task in advance. In particular, both the SQT and the sudden turn task in our study required a 180° turn, demanding a similar lower extremity muscle strength and balance but different motor planning processes. Therefore, the training strategies and protocols emphasizing muscle strength and balance as well as the turning-based training in our study improved planned turning performance only and not more demanding tasks, such as sudden turns. A sudden change in path while walking is frequently caused by an obstacle or event that may occur unexpectedly in everyday activities. Further studies are needed to establish effective training protocols that improve ability to perform sudden unexpected turns.

In our previous study, the SQT turn time was most influenced by balance control ability, especially in the anterior and lateral directions. Therefore, in the present study, we designed the specific exercise program accordingly and noted that the improved dynamic balance performance, as evidenced by increased Tinetti balance scores and longer maximal excursion in the forward direction on the LOS test, paralleled the improved turning performance. Furthermore, according to Faber *et al*., the minimum detectable changes in Tinetti balance score are 0.7 to 0.8 in elderly populations^20^. The change values in Tinetti balance score in our specific exercise group were 1.3 at post-training and 0.9 at follow-up, which were both greater than 0.8. When turning, people must move their COG forward to the targeted side and take asymmetric steps to redirect themselves while maintaining dynamic balance^31^. The improved maximal excursion suggests that this intervention may reduce dyskinesia by improving the ability to select effective sway strategies and manage the controlled movement^32^. Specifically, the improved dynamic balance control increased the potential ability to effectively perform turning tasks. However, the results of the LOS test for the lateral sides, which also affect turning performance as noted in our previous study, showed no significant improvements after training. The anterior margin of stability is reduced to a greater extent than the lateral margin of stability in patients with PD^33^,^34^, which was also confirmed in this study in the baseline measurements. Previously, we found greater improvements in the anterior direction than in the lateral direction for the LOS test after 12 sessions of balance and strengthening exercises in patients with PD^8^. Therefore, training programs that involve greater intensity or frequency may result in better lateral balance control ability. However, most of our dynamic exercise tasks, for instance, reaching, ring tossing, and catching/throwing a ball, focused mostly on forward and not lateral direction control. Therefore, the greater improvements in the anterior direction during the LOS test may also be due to the specificity of the tasks being trained.

In addition to balance control ability, lower extremity muscle strength also plays an important role in turning tasks^6^. When turning, lower extremity extensors are essential for transferring weight, whereas the hip abductors are important for medial-lateral stability. Furthermore, ankle dorsiflexors also have a substantial influence on balance^35^,^36^. We found that the lower extremity extensors, abductors, and ankle dorsiflexors were the most responsive muscles to our 12-session specific exercise training program. Therefore, increases in the strength of these muscles contribute to an improved ability to move the legs toward and away from the center of the body, which may lead to superior turning performance. However, in light of a meta-analysis review, progressive resistance training programs may significantly increase lower extremity muscle strength but not walking ability in people with PD^37^. Progressive resistance training must thus include task-specific function or balance training to generate functional improvements. Therefore, the improvements in turning ability demonstrated in our study may have resulted not only from the strengthening exercises but also from the balance and turning activity training included in the specific exercise group.

Although the turning-based training did not exert better effects on the above mentioned muscle groups than the specific exercise group, it exerted better effects on the ankle plantar flexors. The plantar flexors are necessary for propulsion during turning and can be strengthened by undergoing turning-based treadmill training for at least 12 sessions. Moreover, our turning-based training exerted beneficial effects on vestibular integration ability. People require vestibular input to prepare to turn, and vestibular feedback helps control axial movements while rotating the head and trunk during turning^38^. Compared to healthy age-matched controls, PD patients suffer from balance disorders due to decreases in their ability to process visual and vestibular inputs^39^. Vestibular function may have been stimulated and improved through walking on the turning-based treadmill. Such improvements have also been documented in patients with stroke^11^. Thus, the positive effects on vestibular function together with improved muscle strength may result in better turning performance in individuals with PD after turning-based training.

As demonstrated by the 1-month follow-up data, participants receiving balance and muscle strengthening and participants receiving turning-based training followed by turning training on a level surface retained the effects of training. Therefore, the training strategies proposed above are recommended to enhance turning performance in individuals with PD.

The major limitation of this study was the relatively small sample size. A larger randomized, controlled clinical trial is needed to validate the reported benefits of the specific exercise and turning-based treadmill training programs. Despite the small sample size, the effect size was relatively strong for the outcomes (turn time, η^2^ = 0.21). In addition, the therapist was not blinded to group assignment; although unavoidable, this limitation may have introduced bias. It should also be noted that regular treadmill training is an effective intervention for improving gait performance in individuals with PD; therefore, the effects of treadmill training cannot be ruled out as influencing the improvements demonstrated in the turning-based treadmill training group. Moreover, it should be noted that all participants in our three groups received 10 minutes of ground turning training after 30 minutes of the primary training program to enhance the effects of the exercise. However, this may have blurred the distinction between the three groups.

In summary, difficulty turning is a major problem in Parkinson’s disease. However, there is a lack of effective training programs that improve turning performance. Based on our results, turning performance can be improved by balance and muscle strengthening and by turning-based treadmill training following turning training on a level surface. Therefore, these two types of training programs can be adopted in the clinical rehabilitation of the PD population to improve their turning performance and possibly decrease their risk of falls.

In conclusion, we propose two different training strategies to improve turning performance in individuals with PD: a specific exercise program emphasizing balance and muscle strengthening training and a turning-based treadmill training program. However, the improvements identified in turning performance in these two groups accompanied improvements in different aspects of impairment. Furthermore, the improvements persisted for at least 1 month after training.

## Methods

### Participants

The participants were recruited from medical centers in Taiwan and diagnosed with idiopathic PD by a neurologist. The diagnostic criteria for PD were the presence of at least two of four features (resting tremor, bradykinesia, rigidity, and asymmetric onset), one of which had to be resting tremor or bradykinesia^40^. To be included in this study, participants with PD had to satisfy the following criteria: (a) Hoehn and Yahr stage of I to III, (b) ability to walk independently, (c) stable medication usage, and (d) a score of at least 24 on the mini-mental state examination (MMSE). The exclusion criteria were as follows: (a) unstable medical condition and (b) history of other neurological, cardiopulmonary, or orthopedic disease known to interfere with participation in the study. Written informed consent was obtained from all subjects. The study protocol was approved by the Institutional Review Boards of Taipei City Hospital, Chang Gung Medical Foundation, and Mackay Memorial Hospital. The methods were carried out in accordance with the approved guidelines. This trial was registered at http://www.anzctr.org.au/ (ACTRN12616000198426 on February 15, 2016) and conformed to the CONSORT flowchart (Fig. 1) and checklist.

### Experimental design

This study was a single-blinded, parallel randomized, controlled trial. A research assistant in the medical center enrolled the participants. An individual who was not involved in the study generated the random allocation sequence and selected one of a set of sealed envelopes to assign participants to one of three groups (block randomization; allocation ratio = 1): the specific exercise group, the turning-based training group, and the control group. Participants in each group received one-on-one sessions comprising 30 minutes of specific exercise, turning-based treadmill training, or control exercise, depending on their assignment, followed by 10 minutes of turning/walking training on a level surface for a total of 12 sessions over a 4–6-week period. All training procedures were implemented under a physical therapist’s supervision. All outcomes were measured the day before the intervention (pre), the day after completing the intervention (post), and on the 30th day after completing the intervention (follow-up) by the same rater who was blinded to group assignment. The measurements and interventions were conducted with patients in the “on” state. Before each training session, the participants were informed of the potential adverse effects and discomfort (i.e., dizziness, falls, or muscle soreness).

### Interventions

#### Specific exercise training

Our previous study found that balance ability and lower extremity muscle strength were significantly correlated with turning performance in individuals with PD^6^. Therefore, participants in the specific exercise group received specific exercise training that included 20 minutes of balance exercises and 10 minutes of muscle strengthening exercises in each session as described below.

- Balance exercises: The balance exercises focused on both static and dynamic standing balance training. For instance, standing on ground composed of different materials and bases of support was a treatment exercise to train static balance. For dynamic balance training, catching/throwing a ball, the ring toss game, and forward reaching in different directions were included. The progressions in balance training depended on an individual’s abilities and included decreases in support base and increases in movement range or movement speed, etc. Please refer to the Supplementary Table S1 for details.
- Strengthening exercises: The strengthening training focused on the hip extensors, abductors, and knee extensors, which have been highly correlated with turning performance^6^. Training intensity started at 50% of maximal voluntary contraction (MVC) for 10 repetitions and increased progressively to 75%–80% of MVC. After the individual muscle concentric strengthening exercises, participants performed squats, stepping up/down in the standing position, and bridging and hip circle activities in the supine position. Normal breathing was emphasized during the training session.

#### Turning-based training

Participants in the turning-based training group received turning training on a rotational treadmill. This rotational treadmill (Rmax Science & Technology Co., Ltd., New Taipei City, Taiwan) was designed to provide turning-based (task-specific) training. The basic design of the rotational treadmill was similar to a regular treadmill, with the exception of its circular, running motor belt. The small radius (0.8 m) forced participants to perform a turning action rather than walk straight. In a previous study, we demonstrated that this type of turning-based treadmill training was feasible and effective in improving turning ability in subjects with chronic stroke^11^.

Participants were trained in both directions, with one leg as the inner limb for 15 minutes and the same leg as the outer limb for another 15 minutes, as the treadmill rotated either clockwise or counterclockwise. During the training period, all participants wore a safety harness without body weight support to prevent possible falls. The participants could place their hands on the handrail to support their balance; however, they were encouraged to refrain from holding the handrail. A therapist provided verbal feedback to encourage the participants to walk with a large stride and correct posture. A gradual increase in speed is considered the most important factor for treadmill training to challenge the walking ability of PD patients^41^. Hence, the treadmill speed in the present study, set at 80% of participants’ comfortable turning speed on level ground at the start of training, was increased by 0.05 m/s every 5 minutes as tolerated. Maintenance of an upright posture and a perceived exertion of “somewhat hard” or lower (i.e., a Borg rating of perceived exertion <13) were two criteria for increasing training speed^42^.

#### Control exercise training

Participants in the control group received 30 minutes of trunk exercises combining upper limb movements in the sitting position that minimally challenged their standing balance and lower extremity muscle strength. These exercises included trunk flexion, extension, rotation, and side bending with different arm movements (i.e., shoulder flexion, extension, abduction, rotation, and diagonal lifting and chopping).

The training intensity of the 3 groups was maintained at a perceived exertion of “hard” (a Borg rating of perceived exertion <15)^43^,^44^. The criteria for training progression were determined by the ability of the participant to perform the activities without difficulty and by their perceived exertion (Borg rate of perceived exertion <13, “somewhat hard”). The training was stopped immediately in the event of any discomfort^42^.

Following the 30-minute exercise training session for each group described above, a 10-minute over-the-ground turning program was implemented. This program included S-shaped, 8-shaped, square-shaped, and oval-shaped turns while walking, with verbal cues for gait correction.

### Outcome measures

#### Primary outcomes

Two different turning tasks were performed to generate the primary outcomes in this study, including the SQT and the sudden 180° turn during walking. All turning tasks were performed toward both sides, three times for each side, and the average of the 6 total times was used for the data analysis. In the SQT, the participants were instructed to take two steps forward on cue, quickly turn 180°, and walk back to the starting position on a long force plate that quantified the vertical forces exerted by the participants’ feet. The turn time was the time needed to execute the 180° turn. The turn sway was used to quantify the postural stability of the subjects during the turn and was expressed as the average center of gravity (COG) sway velocity in degrees/second during the 180° turn^45^.

The sudden 180° turn in the walking test was modified from item 5 of the functional gait assessment (FGA). This assessment has been shown to have good reliability and validity in assessing functional mobility in individuals with PD^46^. We used the same instructions as indicated in the FGA. In this test, the participants were asked to turn 180° as quickly as possible after they were instructed to ‘stop and turn’ while walking along a 10-meter walkway at a comfortable speed. The turn time was measured from the beginning of the instruction to the completion of the 180° turn. The test-retest reliability was 0.93 in 10 healthy subjects.

#### Secondary outcomes

Sensory integration ability, dynamic balance ability, and muscle strength were measured as secondary outcomes in this study.

Sensory integration ability was assessed by the SOT of the Balance Master System. The SOT assesses an individual’s ability to use the three sensory systems (somatosensory, visual, and vestibular) that contribute to postural control under six different visual and support-surface conditions. The equilibrium score was calculated by quantifying the COG sway under the six sensory conditions. In addition, the somatosensory, visual, and vestibular ratios were calculated to indicate the ability to use the somatosensory, visual, and vestibular systems, respectively^45^.

Dynamic balance ability was assessed by the Tinetti balance scale and LOS test of the Balance Master System. The balance section of the Tinetti assessment consists of a 10-item rating of balance performance on tasks, with a higher score indicating better performance^47^,^48^. To assess the LOS, participants stand on a force plate and shift their COG to reach a maximal distance in the target direction as quickly and accurately as possible without moving their feet. The directions assessed included forward and lateral in a random order. Maximum excursion (ME) was obtained during the LOS test in this study. ME was defined as the farthest distance traveled by the COG during the trial.

Muscle strength was evaluated using a handheld dynamometer (Power Track II; Jtech Medical Industries Inc., Herber City, UT, USA). The muscle groups tested were the hip flexors/extensors/abductors/adductors, knee flexors/extensors, and ankle dorsiflexors/plantar flexors. The ‘make’ test method was used, during which the handheld dynamometer was held stationary while the subject exerted a maximal force against it; the testing position for each muscle followed standard protocols^49^. Three contractions of 5 seconds for each muscle group were measured and averaged. The muscle strength of both the hip/knee flexors and hip/knee extensors were summed to indicate the strength of the flexors and extensors of the lower extremities.

### Statistical analysis

Statistical Package for the Social Sciences 20.0 software (IBM Corp. in Armonk, NY, USA) was used for the data analysis. Descriptive statistics were generated for all variables, and the distributions of variables were expressed as the mean ± standard deviation. One-way analysis of variance (ANOVA) or χ2 analysis was used to evaluate the intergroup differences between baseline characteristics. Change values were calculated by subtracting the baseline data from the post-training data or by subtracting the baseline data from the follow-up data. To analyze the intergroup differences, the change values were analyzed using a one-way ANOVA with group as a factor, followed by Tukey’s post hoc test. Because the change values (between post and pre and between follow-up and pre) in the one-way ANOVAs were examined twice, the significance level was corrected with a Bonferroni correction (p = 0.025) to reduce the possibility of statistical error.

The effective sample size in this study was 60, based on the primary outcome effects observed (turning performance) in our previous study^11^. Using an effect size η^2^ of 0.19, a type I error of 0.025, and an 80% power, at least 20 patients in each group were required to identify statistically significant differences in turning performance (one-way ANOVA). However, after recruiting 36 patients, the effect size η^2^ for turning performance was 0.23 and the power was 83%, indicating that the statistical goal had already been reached. Finally, a total of 36 patients were included in the study.

## Additional Information

**How to cite this article**: Cheng, F.-Y. *et al*. Positive Effects of Specific Exercise and Novel Turning-based Treadmill Training on Turning Performance in Individuals with Parkinson’s disease: A Randomized Controlled Trial. *Sci. Rep.* **6**, 33242; doi: 10.1038/srep33242 (2016).

## Supplementary Material

Supplementary Information

## Figures and Tables

**Table 1:** Baseline demographic and clinical characteristics of participants.

	Specific exercise group (n = 12)	Turning-based training group (n = 12)	Control group (n = 12)	P value
Age (years)	66.4 ± 7.8	65.8 ± 11.5	67.3 ± 6.4	0.828
Gender (male/female)	8/4	9/3	8/4	0.880
Height (cm)	159.6 ± 5.4	162.8 ± 6.6	163.3 ± 10.8	0.345
Weight (kg)	61.5 ± 6.7	68.0 ± 12.5	63.4 ± 16.0	0.429
MMSE	28.1 ± 1.8	27.7 ± 1.3	28.1 ± 1.1	0.987
Hoehn-Yahr Stage	1.6 ± 0.8	1.8 ± 0.6	2.0 ± 0.8	0.308
1–1.5	4	5	5	
2–2.5	6	5	4	
3	2	2	3	
Onset duration (years)	6.5 ± 2.4	6.1 ± 4.1	8.1 ± 4.6	0.371
Comfortable gait speed (cm/s)	78.2 ± 19.5	82.9 ± 24.9	74.9 ± 24.8	0.698

MMSE, mini-mental state examination.

**Table 2:** Comparisons of turning performance.

	Specific exercise group (n = 12)			Turning-based training group (n = 12)			Control group (n = 12)		
	Pre	Post	Follow up	Pre	Post	Follow up	Pre	Post	Follow up
Step/quick turn (SQT)									
Turn time (s)	2.7 ± 2.7	1.8 ± 1.7	2.2 ± 2.4	2.6 ± 2.0	1.7 ± 1.3	1.9 ± 1.8	2.7 ± 0.5	2.7 ± 0.6	2.6 ± 0.7
Change values^a^		−0.9 ± 1.0*	−0.5 ± 0.4		−0.9 ± 1.0*	−0.6 ± 0.8		0.1 ± 0.5	0.0 ± 0.6
Turn sway (°)	39.1 ± 11.8	32.5 ± 11.5	36.2 ± 15.1	41.5 ± 14.0	32.5 ± 11.5	36.2 ± 15.1	34.7 ± 8.0	35.1 ± 8.3	34.0 ± 9.1
Change values^a^		−7.7 ± 7.6	−5.0 ± 5.9		−9.0 ± 12.9	−5.3 ± 12.0		0.3 ± 7.6	0.7 ± 8.8
Sudden 180° turn during walking									
Turn time (s)	3.8 ± 3.1	3.2 ± 2.4	3.4 ± 2.7	3.4 ± 1.4	2.8 ± 0.9	3.0 ± 1.2	3.2 ± 1.2	3.0 ± 1.1	3.3 ± 1.2
Change values^a^		−0.7 ± 0.8	−0.4 ± 0.5		−0.9 ± 0.8	−0.5 ± 0.3		−0.2 ± 0.6	0.1 ± 0.4

^a^Change values were calculated by subtracting the baseline data from the post-training data (post) or by subtracting the baseline data from the follow-up data (follow-up). *p < 0.025 for intergroup comparis ons (vs. control group).

**Table 3:** Comparisons of SOT score.

	Specific exercise group (n = 12)			Turning-based training group (n = 12)			Control group (n = 12)		
	Pre	Post	Follow up	Pre	Post	Follow up	Pre	Post	Follow up
Total score	71.0 ± 6.0	75.2 ± 5.7	76.1 ± 5.7	71.8 ± 6.1	78.9 ± 4.2	78.3 ± 5.9	70.2 ± 7.4	71.1 ± 7.3	70.4 ± 8.1
Change values^a^		4.2 ± 2.7	5.1 ± 3.2		7.1 ± 4.2*	6.6 ± 1.9		0.9 ± 4.2	2.2 ± 4.7
Somatosensory ratio	0.95 ± 0.03	0.97 ± 0.02	0.96 ± 0.02	0.96 ± 0.03	0.95 ± 0.04	0.95 ± 0.04	0.97 ± 0.02	0.98 ± 0.02	0.98 ± 0.02
Change values^a^		0.01 ± 0.04	0.01 ± 0.03		−0.06 ± 0.02	−0.05 ± 0.02		0.01 ± 0.02	0.01 ± 0.03
Visual ratio	0.88 ± 0.05	0.89 ± 0.08	0.90 ± 0.08	0.87 ± 0.08	0.90 ± 0.07	0.90 ± 0.06	0.84 ± 0.08	0.89 ± 0.04	0.89 ± 0.06
Change values^a^		0.01 ± 0.04	0.02 ± 0.04		0.03 ± 0.03	0.03 ± 0.05		0.05 ± 0.09	0.04 ± 0.05
Vestibular ratio	0.58 ± 0.18	0.59 ± 0.11	0.60 ± 0.11	0.50 ± 0.15	0.69 ± 0.13	0.63 ± 0.13	0.52 ± 0.20	0.55 ± 0.14	0.55 ± 0.16
Change values^a^		0.01 ± 0.15	0.02 ± 0.13		0.19 ± 0.16*^†^	0.14 ± 0.14^†^		0.04 ± 0.12	0.03 ± 0.11

^a^Change values were calculated by subtracting the baseline data from the post-training data (post) or by subtracting the baseline data from the follow-up data (follow-up). *p < 0.025 for intergroup comparisons (vs. control group). ^†^p < 0.025 for intergroup comparisons (vs. specific exercise group).

**Table 4:** Comparisons of Tinetti balance score and limits of stability (LOS).

	Specific exercise group (n = 12)			Turning-based training group (n = 12)			Control group (n = 12)		
	Pre	Post	Follow up	Pre	Post	Follow up	Pre	Post	Follow up
Tinetti assessment scale									
Balance score	13.8 ± 3.9	15.0 ± 3.2	14.7 ± 3.4	13.3 ± 3.3	14.1 ± 2.8	13.8 ± 3.1	14.1 ± 2.0	14.2 ± 1.7	14.0 ± 1.8
Change values^a^	1.3 ± 1.1*	0.9 ± 1.2		0.8 ± 0.9	0.6 ± 0.7		0.1 ± 0.5	−0.1 ± 0.7	
360° turn time (s)	5.4 ± 2.9	3.9 ± 1.5	4.3 ± 1.8	5.7 ± 3.9	3.8 ± 2.9	4.3 ± 3.2	5.1 ± 2.6	4.8 ± 1.9	5.6 ± 2.2
Change values^a^	−1.7 ± 1.6*	−1.2 ± 1.4*		−2.0 ± 1.7*	−1.5 ± 1.5*		0.2 ± 0.6	0.5 ± 1.5	
LOS: Maximal excursion (%)									
Anterior	52.0 ± 19.0	65.4 ± 15.6	62.0 ± 15.7	54.6 ± 27.8	58.6 ± 17.5	60.7 ± 21.7	49.1 ± 12.5	49.3 ± 11.6	45.9 ± 11.2
Change values^a^	13.4 ± 12.2	10.0 ± 17.2*		4.0 ± 16.1	6.1 ± 12.9		0.2 ± 8.8	−3.2 ± 12.1	
Lateral	71.5 ± 17.6	81.0 ± 17.8	76.0 ± 15.8	73.3 ± 17.2	77.3 ± 13.8	75.0 ± 14.8	66.1 ± 15.2	71.4 ± 12.1	72.0 ± 12.2
Change values^a^	9.9 ± 7.1	4.9 ± 4.2		6.5 ± 10.0	4.0 ± 7.8		4.5 ± 7.9	5.9 ± 7.5	

^a^Change values were calculated by subtracting the baseline data from the post-training data (post) or by subtracting the baseline data from the follow-up data (follow-up). *p < 0.025 for intergroup comparisons (vs. control group).

**Table 5:** Comparisons of muscle strength.

	Specific exercise group (n = 12)			Turning-based training group (n = 12)			Control group (n = 12)		
	Pre	Post	Follow up	Pre	Post	Follow up	Pre	Post	Follow up
LEf (N)	527.6 ± 158.4	567.4 ± 158.5	543.4 ± 146.3	539.9 ± 239.5	585.9 ± 233.0	558.1 ± 246.2	533.8 ± 118.9	519.4 ± 95.3	503.8 ± 102.6
Change values^a^		39.8 ± 86.1	15.8 ± 75.3		47.3 ± 50.6	19.3 ± 46.7		−14.4 ± 34.4	−34.6 ± 47.5
LEe (N)	539.1 ± 152.6	659.6 ± 188.9	628.1 ± 183.0	535.9 ± 237.8	591.3 ± 237.4	563.3 ± 238.7	549.0 ± 122.5	553.3 ± 107.2	525.2 ± 102.3
Change values^a^		120.5 ± 59.12**^†^	89.0 ± 83.7**		55.4 ± 61.2	29.0 ± 83.7		4.2 ± 33.9	−24.6 ± 36.3
LEabd (N)	338.1 ± 117.9	433.0 ± 146.3	393.0 ± 130.2	343.1 ± 165.7	376.0 ± 155.5	352.9 ± 157.5	350.9 ± 120.0	350.4 ± 104.0	325.3 ± 87.0
Change values^a^		94.9 ± 73.1**^†^	54.9 ± 46.0**^†^		33.7 ± 29.5	11.1 ± 19.6		−0.1 ± 27.8	−25.6 ± 52.9
LEadd (N)	208.7 ± 68.1	231.6 ± 70.6	220.2 ± 68.8	217.8 ± 96.7	231.2 ± 90.4	223.9 ± 91.0	214.9 ± 42.8	211.1 ± 39.0	204.0 ± 39.0
Change values^a^		23.0 ± 46.0	10.7 ± 34.1		14.9 ± 19.4	8.6 ± 15.1		−3.8 ± 13.1	−10.9 ± 19.6
Ad (N)	217.1 ± 67.4	238.9 ± 53.9	232.1 ± 54.6	217.1 ± 76.7	226.2 ± 72.0	218.3 ± 70.3	213.8 ± 93.6	205.7 ± 83.1	181.0 ± 73.3
Change values^a^		21.8 ± 51.6	15.0 ± 46.8*		9.1 ± 25.8	0.4 ± 22.1		−8.1 ± 28.3	−32.8 ± 31.9
Ap (N)	216.5 ± 65.2	239.9 ± 65.3	213.7 ± 49.3	220.2 ± 83.7	254.9 ± 74.5	237.0 ± 76.0	219.8 ± 68.4	214.8 ± 62.1	204.2 ± 59.0
Change values^a^		23.4 ± 44.1	−1.2 ± 24.9		33.0 ± 25.8*	16.7 ± 21.6*		−5.0 ± 23.7	−15.7 ± 17.5

LEf, lower extremity flexors; LEe, lower extremity extensors; LEabd, lower extremity abductors; LEadd, lower extremity adductors; Ad, ankle dorsiflexors; Ap, ankle plantar flexors. ^a^Change values were calculated by subtracting the baseline data from the post-training data (post) or by subtracting the baseline data from the follow-up data (follow-up). *,**p < 0.025, p < 0.001, respectively, for intergroup comparisons (vs. control group). ^†^p < 0.025 for intergroup comparisons (vs. specific exercise group).
