# Supplementary material for: Positive Effects of Specific Exercise and Novel Turning-based Treadmill Training on Turning Performance in Individuals with Parkinson’s disease: A Randomized Controlled Trial
Source: Sci Rep. 2016 Sep 13;6:33242. doi: 10.1038/srep33242 (PMC5023848; doi:10.1038/srep33242)
Supplement: Supplementary Information [file srep33242-s1.pdf]

Positive Effects of Specific Exercise and Novel Turning-based Treadmill Training on Turning Performance in Individuals with Parkinson's disease: A Randomized Controlled Trial

Fang-Yu Cheng, PT, PhD[1], Yea-Ru Yang, PT, PhD[1], Li-Mei Chen, MD[2], Yih-Ru Wu, MD[3], Shih-Jung Cheng, MD[4], and Ray-Yau Wang, PT, PhD[1,*]

[1]Department of Physical Therapy and Assistive Technology, National Yang-Ming University, Taipei, 112, Taiwan

[2]Department of Neurology, Cheng Hsin General Hospital, Taipei, 112, Taiwan

[3]Department of Neuromuscular Disorder, Chang Guan Memorial Hospital, Linkou, 333, Taiwan

[4]Department of Neurology, Mackay Memorial Hospital, Taipei, 104, Taiwan

*Corresponding author:

Ray-Yau Wang, PT, PhD, Department of Physical Therapy and Assistive Technology, National Yang-Ming University, 155, Sec 2, Li Nong St., Shih-Pai, Taipei, 112, Taiwan

Tel: 886-2-28267210

FAX: 886-2-28201841

Email: rywang@ym.edu.tw

Balance exercise program	Duration	Progression	Motor demand
Standing on the ground	30 seconds/session	Different materials<br>Reduced bases of support	Static balance
Dynamic standing weight shift	10 minutes/session	Speed<br>Moving range<br>Combined trunk and arm movement	Dynamic balance<br>Weight shifting<br>Coordination
Catching and throwing a ball in standing	20 times/session	Ball size and weight<br>On ground or on rock board	Weight shifting<br>Dynamic balance<br>Coordination
The ring toss game in standing	20 times/session	Toss distance	Dynamic balance<br>Coordination
Reaching activity in standing	20 times/session	Reaching distance<br>Reaching direction	Dynamic balance

Supplementary Table S1. Balance exercise protocol for the specific exercise group.
